# The antioxidant xanthorrhizol prevents amyloid-β-induced oxidative modification and inactivation of neprilysin

**DOI:** 10.1042/BSR20171611

**Published:** 2018-02-02

**Authors:** Chol Seung Lim, Jung-Soo Han

**Affiliations:** 1 Center for Neurodegenerative Diseases, Blanchette Rockefeller Neurosciences Institute, West Virginia University, Morgantown, WV 26505, U.S.A.; 2 Department of Biological Sciences, Konkuk University, Seoul 05029, South Korea

**Keywords:** amyloid-β, anti-oxidant, HNE, neprilysin, oxidative stress, xanthorrhizol

## Abstract

Activity of neprilysin (NEP), the major protease which cleaves amyloid-β peptide (Aβ), is reportedly reduced in the brains of patients with Alzheimer’s disease (AD). Accumulation of Aβ generates reactive oxygen species (ROS) such as 4-hydroxynonenal (HNE), and then reduces activities of Aβ-degrading enzymes including NEP. Xanthorrhizol (Xan), a natural sesquiterpenoid, has been reported to possess antioxidant and anti-inflammatory properties. The present study examined the effects of Xan on HNE- or oligomeric Aβ_42_-induced oxidative modification of NEP protein. Xan was added to the HNE- or oligomeric Aβ_42_-treated SK-N-SH human neuroblastoma cells and then levels, oxidative modification and enzymatic activities of NEP protein were measured. Increased HNE levels on NEP proteins and reduced enzymatic activities of NEP were observed in the HNE- or oligomeric Aβ_42_-treated cells. Xan reduced HNE levels on NEP proteins and preserved enzymatic activities of NEP in HNE- or oligomeric Aβ_42_-treated cells. Xan reduced Aβ_42_ accumulation and protected neurones against oligomeric Aβ_42_-induced neurotoxicity through preservation of NEP activities. These findings indicate that Xan possesses therapeutic potential for the treatment of neurodegenerative diseases, including AD, and suggest a potential mechanism for the neuroprotective effects of antioxidants for the prevention of AD.

## Introduction

Alzheimer’s disease (AD) is a progressive and irreversible neurodegenerative disorder characterized by cognitive decline [1,2]. AD is also characterized morphologically by extensive amyloid deposition, neurofibrillary tangles, and neuroinflammation leading to synaptic and neuronal loss [3,4]. Oligomeric forms of amyloid-β peptide (Aβ) aggregate and form senile plaques in the brains of patients with AD [5,6]. Abnormal accumulation of Aβ is toxic to neurones [7,8]. Hence, treatments that prevent Aβ accumulation could slow the neurodegeneration and cognitive decline in AD.

Increasing evidence has shown that Aβ accumulation enhances its production and decreases its degradation through the actions of several Aβ-degrading enzymes, including neprilysin (NEP), insulin-degrading enzyme, and endothelin-converting enzyme [9–12]. NEP, a predominant Aβ protease, cleaves both monomeric and oligomeric forms of Aβ in the brain [13–15]. NEP protein levels are reduced in the hippocampus and cortex of aged mice [16,17], and NEP is selectively down-regulated in areas of the AD brain with high levels of amyloid plaques [18,19]. Conversely, overexpression of NEP reduces Aβ levels in a dose-dependent manner [20,21], and protects neurones from Aβ-induced toxicity *in vitro* [22]. These results imply an inverse correlation between NEP activity and Aβ levels, supporting the hypothesis that a reduction in NEP expression or its activity induces Aβ deposition, and the subsequent neuronal dysfunction occurs in AD.

Oxidative stress has long been recognized as an important factor in the early development of AD [23–25]. Aβ induces high levels of reactive oxygen species (ROS) [26]. Specifically, elevated levels of 4-hydroxynonenal (HNE), an α,β-unsaturated hydroxyalkenal that is produced by lipid peroxidation in Aβ deposits could interact with, modify, and inactivate a variety of cellular proteins and enzymes [27,28]. It has been reported that NEP is modified by HNE and catalytic activity of HNE-modified NEP is decreased in AD brains and in HNE- or Aβ-treated cells [29,30]. Therefore, it is expected that prevention of NEP oxidative modification may increase NEP activity and increased NEP activity may reduce Aβ accumulation, which in turn results in protection of neurones against Aβ-induced neurotoxicity.

Antioxidants have been reported as promising treatments for protecting neurones against oxidative stress [31,32]. Xanthorrhizol (Xan), isolated from *Curcuma xanthorrhiza* RoxB, has been reported to possess antibacterial and anti-inflammatory activity [33]. It is also reported that Xan has antioxidant properties, i.e. it directly scavenges hydrogen peroxide, it prevents ROS production and ROS-induced cell death, and it inhibits oxidative damage by reducing lipid peroxidation of cellular proteins [34]. Therefore, the present study examined effects of Xan on the oxidative NEP modification and NEP activities in HNE- or oligomeric Aβ_42_-treated neuroblastoma cells, along with N-acetyl-l-cysteine (NAC) that has been reported to reduce Aβ_42_-mediated oxidative modification [35].

## Materials and methods

### Cell culture and treatment

Human neuroblastoma SK-N-SH cells were obtained from the American Type Culture Collection (ATCC, HTB-11, Manassas, VA) and maintained in essential medium supplemented with 1 µM non-essential amino acids, 100 UI/ml penicillin, 100 µg/ml streptomycin, and 10% (v/v) FBS (all culture materials from Invitrogen, Carlsbad, CA) in a humidified atmosphere with 5% CO_2_ at 37°C. Cells were subcultured twice per week and had undergone four to eight passages prior to the experiments.

A combination of HNE (Cayman Chemical, Ann Arbor, MI), Xan (Enzo Life Sciences, Farmingdale, NY), NAC (Sigma–Aldrich, St. Louis, MO), oligomeric Aβ_42_ (AnaSpec, Fremont, CA), or thiorphan (TP, Cayman Chemical), a specific NEP inhibitor, was added to the cultured cells according to experimental design. Xan and NAC were dissolved and diluted in Dulbecco’s PBS (DPBS, pH 7.4). HNE and TP were freshly prepared in DMSO and diluted in PBS prior to the experiment. To induce oxidative modification of NEP, cells were kept in 2% serum-reduced medium for 16 h, and HNE (10 µM) or oligomeric Aβ_42_ (1 µM) were then added to the cultured cells for 12 h. The same volume PBS was added to the cultures to serve as untreated controls.

### Preparation of Aβ_42_

Monomeric and oligomeric Aβ_42_ were prepared as described previously [36], from aliquots of the same batch of Aβ_42_. For oligomeric Aβ_42_, lyophilized Aβ_42_ aliquots (0.3 mg) were dissolved in 0.2 ml of 1,1,1,3,3,3-Hexafluoro-2-propanol (HFP, Sigma–Aldrich) and then added to 0.7 ml H_2_O. Samples were loosely capped and stirred on a magnetic stirrer under a fume hood for 48 h, and used within 36 h. Monomeric Aβ_42_ was prepared immediately before use by rapidly evaporating the HFP via gentle bubbling of nitrogen gas into the solution. The quality of Aβ_42_ preparations was checked by immunoblot with anti-A-11 (1:1000, Invitrogen) and anti-6E10 (1:1000, Covance, Princeton, NJ) antibodies.

### Immunoprecipitation and immunoblot analysis

Cultured cells were lysed in cold lysis buffer (10 mM Tris/HCl, pH 7.4, 5 mM EDTA, 1% Triton X-100, 10% glycerol, 1 mM CaCl_2_, 1 mM MgCl_2_, and 1× complete protease inhibitor cocktail (Thermo Scientific, Waltham, MA)) for 1 h at 4°C. Total lysates (1 mg) were immunoprecipitated with an anti-NEP antibody (1 µg/ml, Abcam, Cambridge, MA) at 4°C overnight, and protein/antibody immunocomplexes were purified with protein A-magnetic beads and a magnetic separator (both from Millipore, Temecula, CA). After washing, immunocomplexes were separated by SDS-PAGE using 10% gels (Invitrogen), transferred on to nitrocellulose membranes, and incubated with the primary rabbit polyclonal anti-HNE or rabbit polyclonal anti-NEP (both from Millipore) antibodies at room temperature overnight. After incubation with the secondary horseradish peroxidase (HRP)-conjugated goat anti-rabbit or mouse IgG (1:10000; Jackson ImmunoResearch Lab, West Grove, PA) antibodies, the membranes were developed with ECL and exposed to X-ray film (Thermo Scientific). As a control, membranes were stripped and re-probed with the primary mouse anti-NEP (1:1000, Abcam) or mouse anti-β-actin (1:4000, Sigma–Aldrich) antibodies followed by the secondary HRP-conjugated goat anti-mouse IgG (1:10000, Jackson ImmunoResearch Lab) antibody. Immunoreactivity was assessed by densitometric analysis of films using an HP Scanjet densitometer (Hewlett-Packard, Corvallis, OR) and ImageJ image analysis software (1.47v, NIH, Bethesda, MD) as described previously [37].

### Immunocytochemistry

Cells grown on 2% gelatin (Sigma–Aldrich) coated coverslips (Carolina Biological Supply Company, Burlington, NC) were fixed with 4% paraformaldehyde at room temperature for 20 min and permeabilized with 0.2% Triton X-100 in 1× PBS (pH 7.4). After pre-blocking for 1 h at room temperature with 1% normal goat serum/1× PBS, cells were incubated overnight at 4°C in a humidified chamber with the primary mouse anti-NEP (1:100, Abcam) or rabbit anti-HNE (1:200, Millipore) antibodies. At the end of the incubation period, the cells were rinsed three times with 1× PBS containing 0.05% Tween-20 (PBS-T) and then incubated with the secondary Alexa 488-conjugated goat anti-mouse or Alexa 568-conjugated goat anti-rabbit IgG (1:500, Invitrogen) for 60 min at room temperature. All primary and secondary antibodies were diluted in PBS-T with 2% normal goat serum. After rinsing with 1× PBS, the coverslips were mounted using ProLong Gold antifade reagent with DAPI (Invitrogen), and viewed and photographed on a Zeiss LSM 710 laser scanning confocal microscope (Carl Zeiss, Thornwood, NY). Immunofluorescence staining was repeated at least three times.

### Fluorometric NEP activity assay in cell lysates and intact cells

To determine NEP activity in whole cell lysates, cells were incubated with HNE (10 µM) or oligomeric Aβ_42_ (1 µM) without or with Xan (1, 10 µM) or NAC (10, 100 µM) for 12 h, collected, lysed in 1× PBS (pH 7.4) with 0.1% Triton X-100, and the lysates were placed on ice for 30 min. NEP activity in cell lysates was analyzed using a synthetic NEP fluorogenic peptide substrate (Mca-R-P-P-G-F-S-A-F-K[Dnp]-OH; R&D Systems, Inc., Minneapolis, MN) in the presence/absence of 500 nM TP. Samples were dissolved in 50 mM HEPES buffer (pH 7.5) and pre-incubated with TP or 1× PBS for 20 min prior to adding the NEP fluorogenic peptide substrate (dissolved in HEPES). Fluorescence was read at 320 nm excitation and 405 nm emission on a fluorescent microplate reader (BioTek, Winooski, VT).

The activity of membrane-bound NEP in intact cells was measured as previously described [36]. Briefly, after treatment like above, intact cells were harvested, washed with 1× PBS (pH 7.4), and incubated with 1 mM glutaryl-Ala-Ala-Phe-4-methoxy-2-naphthylamide (Sigma–Aldrich) as the NEP substrate. The substrate solution was collected and incubated with leucine aminopeptidase (50 µg/ml, Sigma–Aldrich, St. Louis, MO, U.S.A.) in the presence/absence of 500 nM TP for 30 min at 37°C, and the released free 4-methoxy-2-naphthylamide was fluorometrically measured at an emission wavelength of 425 nm using a microplate reader (BioTek).

FRET assay was performed for kinetic analyses of NEP activity. Cell lysates prepared by the method described above were incubated with increasing concentrations of NEP fluorogenic peptide substrate (0–20 µM) at room temperature. Fluorescence was measured over a 1-h period. NEP activity was determined as the difference in fluorescence in the presence/absence of 500 nM TP. Kinetic isotherms (*V*_max_ and *K*_m_ values) for NEP activity were determined by non-linear least squares fitting to the Michaelis–Menten equation using GraphPad Prism 6 software (GraphPad Software, Inc., La Jolla, CA).

### *In vitro* Aβ cleavage assay

SK-N-SH cells were incubated with Xan (1, 10 μM) or NAC (10, 100 µM), in the presence/absence of 500 nM TP for 1 h. NEP proteins were isolated by immunoprecipitation from cells. For the *in vitro* cleavage assay, isolated NEP proteins were incubated with the same amount of 2.5 µM monomeric or oligomeric Aβ_42_ in 20 mM HEPES, pH 7.4, 10 mM KCl, and 10 mM MgCl_2_ for an additional 4 h at 30°C. The reaction mixture was separated on SDS/PAGE (10–20% gel) (Invitrogen), blotted, and probed with anti-6E10 antibody (1:1000, Covance) as described in ‘Immunoprecipitation and immunoblot analysis’ section. Cleavage of Aβ_42_ was discerned by the disappearance of protein bands corresponding to intact monomeric and oligomeric Aβ_42_. The densities of the remaining Aβ_42_ bands were quantitated using an HP Scanjet densitometer and ImageJ image analysis software, and plotted using GraphPad Prism 6 software.

### Cell toxicity assay

Cells were cultured in 48- or 96-well plates at a density of ~3000–5000 cells/well in complete growth medium for 24 h. Growth medium was replaced with fresh culture medium (~100–200 µl/well) containing 2.0% FBS and 1 µM oligomeric Aβ_42_ for 24 h. Another batch of cells were co-treated with Xan or NAC in the presence/absence of 500 nM TP. After 24 h, a cell viability assay was performed as described previously [34,36] using the Cell Counting Kit-8 (Dojindo, Rockville, MD). Briefly, ~10–20 µl of CCK-8 solution was added to all wells, and the plates were incubated for 4 h at 37^°^C in 5% CO_2_. The culture medium was collected and detected with a microplate reader at a wavelength of 450 nm. The difference in optical density (OD) relative to untreated controls was taken as a measure of cell viability, and the percentage of viable cells was calculated by comparing the OD at 450 nm for the Aβ_42_-treated and control wells.

### Statistical analysis

Data were expressed with a percentage of untreated controls. All data are presented as mean ± S.E.M. from three or more independent experiments, unless otherwise indicated. Differences between untreated cells and HNE- or oligomeric Aβ_42_-treated cells were examined using a *t* test. One-way ANOVA was conducted to see the effects of Xan and NAC on conditions with these experimental treatments. Subsequent *post hoc* test (Tukey’s multiple comparison) was followed. *P-*values less than 0.05 were considered significant, unless specified otherwise.

## Results

### Xan prevented HNE-induced NEP modification

**Figure 1:**
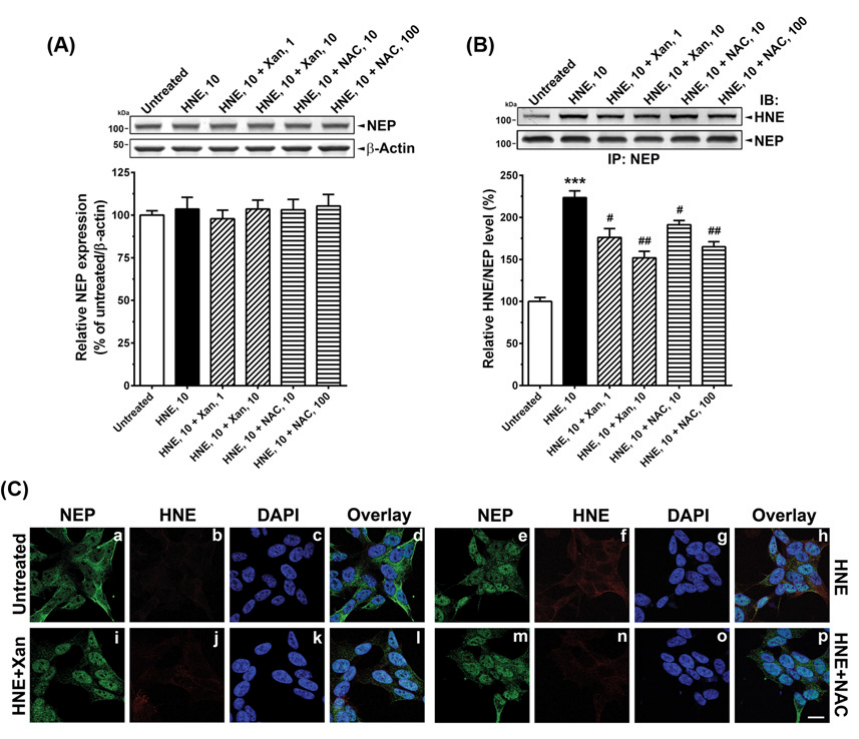
Xan prevents HNE-induced NEP modification HNE-treated (10 µM) SK-N-SH cells were incubated with Xan (1, 10 µM) or NAC (10, 100 µM) and then immunoblot and immunoprecipitation was followed. (**A**) A representative NEP immunoblot (top). Any treatment did not change total protein levels of NEP (bottom). (**B**) Effects of Xan or NAC on the HNE-induced modification of NEP. A representative HNE immunoblot (top). Xan reduced HNE-induced increases in HNE levels on NEP (bottom). (**C**) Untreated cells (a, b, c, d), cells with HNE only (e, f, g, h), cells with HNE and 10 µM Xan (HNE + Xan; i, j, k, l), and cells with HNE and 100 µM NAC (HNE + NAC; m, n, o, p) were immunostained with anti-NEP (green) or anti-HNE (red) antibodies. The nuclei were also stained with DAPI (blue). Cellular localization was determined by confocal overlay imaging. Evidently, Xan reduced HNE-positive signals in HNE-treated cells. Scale bar: 10 µm; mean ± S.E.M. from three independent experiments; ***P<0.001 compared with untreated controls; ^#^*P*<0.05, ^##^*P*<0.01 compared with HNE–treated cells.

Effects of Xan and NAC on the HNE-treated SK-N-SH cells were examined by measurements of oxidative modification of NEP (Figure 1). Any treatment did not change total NEP levels (Figure 1A). However, HNE treatment markedly increased HNE levels on NEP proteins (t(10) =9.73, *P*<0.001; Figure 1B), which was effectively reduced by 1 µM Xan treatment by 38%; 10 µM Xan reduced 59% HNE levels on NEP proteins in the HNE-treated cells (F(2,15) =11.74, *P*<0.01); 10 and 100 µM NAC also reduced 36 and 49% HNE levels on NEP proteins, respectively, in the HNE-treated cells (F(2,15) =11.17, *P*<0.01; Figure 1B).

Furthermore, we observed that HNE-induced HNE increases on NEP proteins using double immunofluorescence staining. As shown in Figure 1C, HNE-positive signals were undetectable in untreated controls, but were abundant and co-localized with NEP-positive signals in the HNE-treated cells. Both 10 µM Xan and 100 µM NAC treatments reduced the HNE-positive signals in the HNE-treated cells.

### Xan prevented oligomeric Aβ_42_-induced HNE modification of NEP

**Figure 2:**
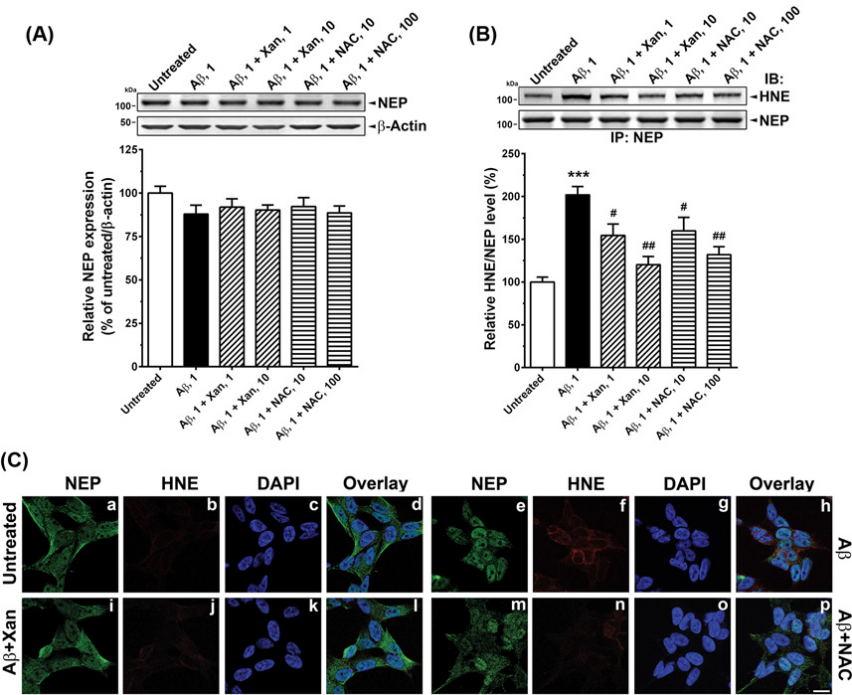
Xan inhibits oligomeric Aβ_42_-induced NEP modification in SK-N-SH cells Aβ_42_-treated (1 µM) SK-N-SH cells were incubated with Xan (1, 10 µM) or NAC (10, 100 µM) and then immunoblotting and co-immunoprecipitation followed. (**A**) A representative NEP immunoblot (top). Any treatment did not change total protein levels of NEP (bottom). (**B**) Effects of Xan or NAC on the Aβ_42_-induced modification of NEP. A representative immunoblot for HNE (top). Xan reduced Aβ_42_-induced increases in HNE levels on NEP (bottom). (**C**) Untreated cells (a, b, c, d), cells with 1 µM oligomeric Aβ_42_ only (e, f, g, h), cells with Aβ_42_ and 10 µM Xan (Aβ + Xan; i, j, k, l), and cells with Aβ_42_ and 100 µM NAC (Aβ + NAC; m, n, o, p) were immunostained with anti-NEP (green) or anti-HNE (red) antibodies. The nuclei were also stained with DAPI (blue). Cellular localization was determined by confocal overlay imaging. Evidently, Xan reduced HNE-positive signals in Aβ_42_-treated cells. Scale bar: 10 µm; mean ± S.E.M. from three independent experiments; ***P<0.001 compared with untreated controls; ^#^*P*<0.05, ^##^*P*<0.01 compared with Aβ_42_-treated cells.

Effects of Xan and NAC on the oligomeric Aβ_42_-treated SK-N-SH cells was examined by measurements of oxidative modification of NEP (Figure 2). Any treatment did not change total NEP levels (Figure 2A). However, oligomeric Aβ_42_ treatments markedly increased HNE levels on NEP proteins (t(10) =9.04, *P*<0.001; Figure 2B); 1 and 10 µM Xan treatments decreased 47 and 75% HNE levels on NEP proteins in the oligomeric Aβ_42_-treated cells, respectively (F(2,15) =11.83, *P*<0.01); 10 and 100 µM NAC treatments also reduced 42 and 70% HNE levels on NEP proteins in the oligomeric Aβ_42_-treated cells, respectively (F(2,15) =8.57, *P*<0.01; Figure 2B).

Furthermore, we observed oligomeric Aβ_42_-induced HNE increases on NEP proteins using double immunofluorescence staining. As shown in Figure 2C, HNE-positive signals were undetectable in untreated controls, but were abundant and co-localized with NEP-positive signals in the oligomeric Aβ_42_-treated cells. Both 10 µM Xan and 100 µM NAC treatments reduced the HNE-positive signals in the oligomeric Aβ_42_-treated cells.

### Xan protected HNE- or oligomeric Aβ_42_-induced NEP inactivation

**Figure 3:**
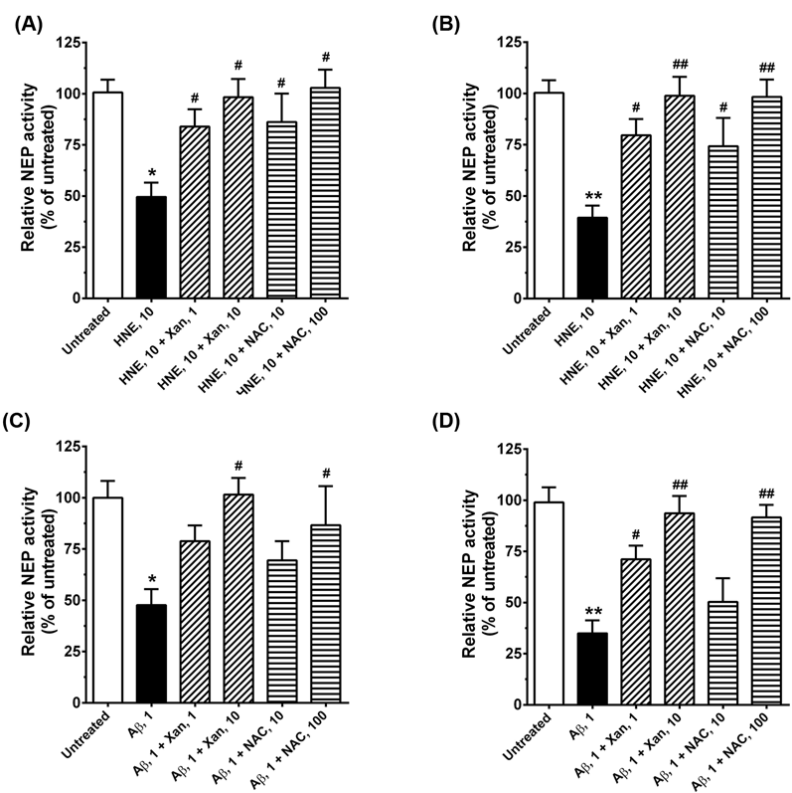
Xan prevents HNE- and oligomeric Aβ_42_-induced reduction in NEP activity in SK-N-SH cells HNE-treated (10 µM) SK-N-SH cells were incubated with Xan (1, 10 µM) or NAC (10, 100 µM) and then NEP activities of whole cell lysates (**A**) and intact cells (**B**) were measured, using a fluorometric peptide substrate. Xan and NAC treatments prevented the HNE-induced reduction in NEP activity. Aβ_42_-treated (1 µM) SK-N-SH cells were incubated with Xan (1, 10 µM) or NAC (10, 100 µM) and then NEP activities of whole cell lysates (**C**) and intact cells (**D**) were measured. Xan and NAC prevented the oligomeric Aβ_42_-induced reduction in NEP activity. Average NEP activity was expressed as a percentage of the untreated control. Mean ± S.E.M. for three independent experiments; *P<0.05, **P<0.01 compared with untreated controls; ^#^*P*<0.05, ^##^*P*<0.01 compared with HNE- or Aβ_42_-treated cells.

HNE treatment induces NEP modification, resulting in inactivation of NEP protein. Therefore, it is predicted that prevention of either HNE- or oligomeric Aβ_42_-induced NEP modification would preserve NEP activity. The present experiment examined the changes in NEP activity in HNE- or oligomeric Aβ_42_-treated SK-N-SH lysates and intact cells, using a fluorometric peptide substrate. Exogenous treatment of 10 µM HNE into cells led to a significant loss of NEP activity, by 51% in whole cell lysates (t(20) =5.45, *P*<0.05; Figure 3A) and by 61% in intact cells (t(20) =7.16, *P*<0.01; Figure 3B). However, 1 and 10 µM Xan treatments increased 35 and 49% NEP activities in the HNE-treated cell lysates, respectively (F(2,30) =9.90, *P*<0.05). In addition, 1 and 10 µM Xan treatments increased 41 and 60% NEP activities in the HNE-treated intact cells, respectively (F(2,30) =16.26, *P*<0.01). Similar patterns were observed in the NAC experiments: 10 and 100 µM NAC treatments increased 37 and 52% NEP activities in the HNE-treated cell lysates, respectively, (F(2,30) =6.81, *P*<0.05; Figure 3A), and 35 and 59% NEP activities in the HNE-treated intact cells, respectively (F(2,30) =8.24, *P*<0.01; Figure 3B).

Since oligomeric Aβ_42_ increases endogenous generation of HNE and subsequently leads to HNE-induced modifications in NEP, we measured NEP activity in oligomeric Aβ_42_-treated cells (Figure 3C,D). Similar to results of HNE-treated cells, oligomeric Aβ_42_ decreased 53% NEP activities in whole cell lysates (t(18) =5.86, *P*<0.05; Figure 3C) and 65% NEP activities in intact cells (t(18) =7.56, *P*<0.01; Figure 3D); 1 and 10 µM Xan treatments recovered 32 and 52% NEP activities in the Aβ_42_-treated whole cell lysates (F(2,27) =11.74, *P*<0.05); 36 and 59% in the Aβ_42_-treated intact cells (F(2,27) =17.05, *P*<0.01); 10 and 100 µM NAC treatments also recovered 23 and 40% NEP activities in the Aβ_42_-treated whole cell lysates, respectively (F(2,27) =3.93, *P*<0.05); 15 and 57% in the Aβ_42_-intact cells, respectively (F(2,27) =9.18, *P*<0.01).

**Figure 4:**
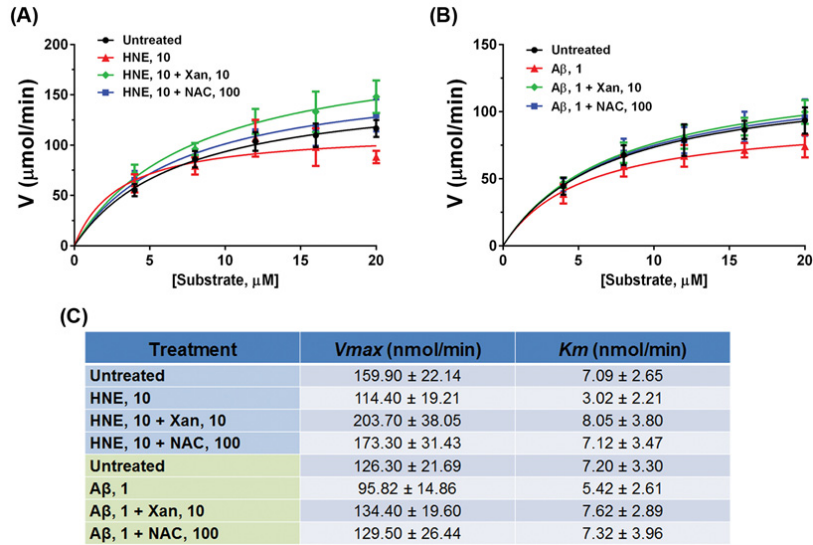
Xan enhances *V*_max_ and *K*_m_ values of NEP enzymatic activity in SK-N-SH cells (**A**) NEP activity in untreated cells, cells with HNE (10 µM), cells with HNE and Xan (10 µM), and cells with HNE and NAC (100 µM) was determined using FRET assay. (**B**) NEP activity in the untreated cells, the cells with oligomeric Aβ_42_ (1 µM), the cells with Aβ_42_ and Xan (10 µM), and cells with Aβ_42_ and NAC (100 µM) was determined using FRET assay. The dependence of the mean NEP activity on increasing substrate concentration (0–20 µM) in SK-N-SH lysates was measured using FRET assay, and then the *V*_max_ and *K*_m_ values (**C**) were calculated. The NEP *K*_m_ values were decreased by HNE or oligomeric Aβ_42_ treatments and preserved by Xan or NAC pre-treatment. Mean ± S.E.M. from three independent experiments.

To further characterize the effect of Xan on NEP activation, we used a range of NEP substrate concentrations (0–20 µM) in SK-N-SH cells and then measured NEP activity using a FRET assay. The measurement of NEP activity was saturable and followed Michaelis–Menten kinetics in all cell samples (Figure 4). The *V*_max_ of NEP activity was decreased in the HNE- or oligomeric Aβ_42_-treated cells; 1 µM Xan pre-treatment cells slightly normalized the *V*_max_ in the HNE- or oligomeric Aβ_42_-treated cells, but it was not statistically significant, compared with the HNE- or oligomeric Aβ_42_-treated cells; however, 10 µM Xan completely recovered the *V*_max_ (Figure 4A,B); 100 µM NAC treatment had a similar effect on the recovery of the *V*_max_ of NEP activity (Figure 4A,B). Lineweaver–Burk double-reciprocal plots of the reaction velocities and substrate concentrations permitted calculation of the Michaelis constant (*K_m_*) for the enzyme in all samples. The NEP *K*_m_ values were decreased by HNE or oligomeric Aβ_42_ treatment and recovered by Xan or NAC pre-treatment (Figure 4C).

### Xan enhanced the ability of NEP to degrade Aβ_42_ peptide and the resistance of SK-N-SH cells to Aβ_42_-induced neurotoxicity through NEP activation

**Figure 5:**
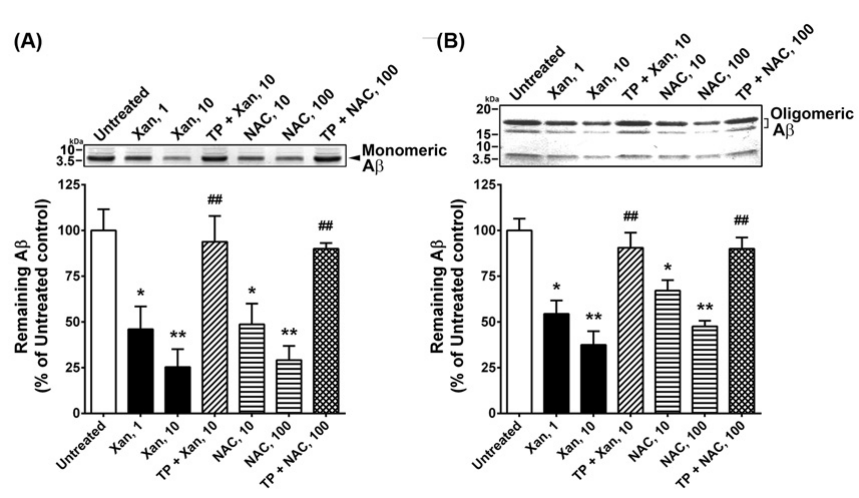
Xan enhances NEP-mediated Aβ_42_ degradation NEP proteins were isolated from untreated cells, cells with Xan (1, 10 µM), cells with TP (500 nM) + Xan (10 µM), cells with NAC (10, 100 µM), and cells with TP + NAC (100 µM), using immunoprecipitation. These isolated NEP proteins were incubated with monomeric (**A**) and oligomeric (**B**) Aβ_42_ (2.5 µM). The densities of the Aβ_42_ bands were quantitated. Xan and NAC decreased monomeric and oligomeric Aβ_42_ levels. However, TP, an NEP inhibitor, abolished the effects of Xan and NAC on the Aβ_42_ degradation. Mean ± S.E.M. for three independent experiments; *P<0.05; **P<0.01 compared with untreated cells; ^##^*P*<0.01 compared with Xan (10 µM)- or NAC (100 µM)-treated cells.

The present experiment examined the effect of Xan though NEP action on the degradation of Aβ_42_; 1 and 10 µM Xan treatments decreased 54 and 72% monomeric Aβ_42_ levels, respectively, compared with the untreated control (F(2,15) =69.57, *P*<0.01). Similarly, 10 and 100 µM NAC treatments decreased 52 and 71% monomeric Aβ_42_ levels, respectively, compared with the untreated control (F(2,15) =74.81, *P*<0.01); 500 nM TP, an NEP inhibitor, almost completely abolished the effect of Xan (t(10) =9.739, *P*<0.01) and NAC (t(10) =13.61, *P*<0.01) on Aβ_42_ degradation (Figure 5A). Notably, 1 and 10 µM Xan treatments cleaved oligomeric Aβ_42_ by approximately 46 and 63%, respectively, compared with the untreated control (F(2,15) =57.96, *P*<0.01). And 10 and 100 µM NAC reduced 37 and 56% oligomeric Aβ_42_ levels, respectively, compared with the untreated control (F(2,15) =47.18, *P*<0.01); 500 nM TP completely abolished the effect of Xan or NAC on oligomeric Aβ_42_ cleavage (Figure 5B).

**Figure 6:**
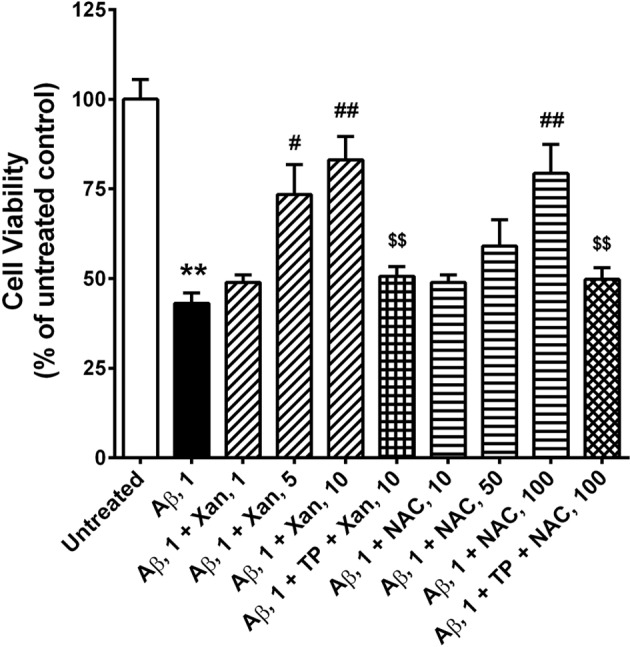
Xan protects against Aβ_42_-induced neuronal toxicity in SK-N-SH cells The viabilities of untreated cells, cells with oligomeric Aβ_42_ (1 µM), cells with oligomeric Aβ_42_ + Xan (1, 5, 10 µM), cells with oligomeric Aβ_4_ + Xan (10 µM) + TP (500 nM), cells with oligomeric Aβ_42_ + NAC (10, 50, 100 µM), and cells with oligomeric Aβ_42_ + NAC (100 µM) + TP, was determined. Viabilities of oligomeric Aβ_42_-treated cells evidently were decreased compared with untreated cells (**). Xan and NAC increased cell viabilities of Aβ_42_-treated cells in a dose-dependent manner, but these Xan and NAC effects was abolished by TP, an NEP inhibitor. Data are expressed as the percentage of untreated cells. Mean ± S.E.M. of three independent experiments; *P*<0.01 compared with untreated controls; ^#^*P*<0.05, ^##^*P*<0.01 compared with Aβ_42_-treated cells; ^$$^*P*<0.01 compared with Aβ_42_ + Xan or Aβ_42_ + NAC-treated cells.

We examined the protective effects of Xan or NAC against oligomeric Aβ_42_-induced neurotoxicity via their NEP action. Oligomeric Aβ_42_-treated SK-N-SH cells were incubated with Xan or NAC in the presence/absence of TP. The viabilities of neuroblastoma cells were significantly decreased by the oligomeric Aβ_42_ treatment (t(14) =9.28, *P*<0.01). But Xan and NAC treatments increased the viabilities of the oligomeric Aβ_42_-treated cells in a dose-dependent manner; 1, 5, and 10 µM Xan increased 5, 30, and 40% viabilities, respectively (F(3,28) =11.94, *P*<0.01), and 10, 50, and 100 µM NAC increased 5, 16, and 36% viabilities (F(3,28) =7.73, *P*<0.01). NEP inhibition by TP almost completely abolished the protective effect of Xan (t(14) =4.63, *P*<0.01) or NAC (t(14) =3.41, *P*<0.01) on cell viability (Figure 6).

## Discussion

During ageing and in neurodegenerative diseases including AD, oxidative stress such as excess reactive HNE results in modification of membrane lipids, DNA, and cellular proteins, which in turn alter their function [2,24,25]. HNE-modified proteins are abundant in the brains of AD patients, suggesting a role of oxidative damage in AD pathogenesis [26,27,35,38]. For example, increased HNE levels on NEP proteins and decreased activities of HNE-modified NEP in Aβ deposits have been observed in the AD brain [30,35]. Experimental evidences have demonstrated that NEP, a major Aβ-degrading enzyme, is one of HNE-induced oxidized proteins [29,30]. Therefore, one of potential strategies for preventing AD is an administration of antioxidants that inhibit HNE-induced NEP modification and prevent the loss of NEP activity [32]. Hence, the present study examined the effects of the antioxidants on HNE- or Aβ-induced NEP modification and activity, and subsequently demonstrated their protective effects through actions on NEP against Aβ_42_-induced neurotoxicity.

Xan is the most active compound isolated from *C. xanthorrhiza* RoxB, possessing several biological activities including antioxidant and anti-inflammatory effects [31]. Specifically, our previous study showed that Xan has anti-inflammatory activity, i.e. it inhibits pro-inflammatory cytokines, such as interleukin-6 and tumor necrosis factor-α, and inhibits nitric oxide (NO) production in lipopolysaccharide-activated microglial cells [34]. In addition, Xan reduces the expression of cyclooxygenase-2 and inducible nitric oxide synthase (iNOS), which results in reduction in NO in activated microglial cells [34]. Xan has potent neuroprotective effects against glutamate-induced neurotoxicity and ROS generation in hippocampal HT22 cells, and inhibits lipid peroxidation in rat brain homogenates with H_2_O_2_ treatments [34]. Therefore, it is expected that Xan, an antioxidant, could inhibit HNE-induced modification of the NEP protein. Both exogenous HNE treatments and the induction of endogenous HNE by oligomeric Aβ_42_ increased HNE levels on NEP proteins. These results are consistent with previous reports that Aβ increased the production of HNE and free radicals in neurones [35,39]. Importantly, Xan reduced HNE levels on NEP proteins in HNE- or Aβ_42_-treated cells. NAC as a positive control showed similar results.

Aβ peptide plays a pivotal role in the pathogenesis of AD [10,12]. Aβ- or oxidant-induced HNE modification reduces the activity of both endogenous and recombinant NEP protein [30,40], following a reduction in Aβ-degrading ability of NEP and Aβ accumulation [29,30]. It is also confirmed in our previous study that the activity of NEP was reduced in oligomeric Aβ_42_-treated cells [36]. Thus, reduction in NEP activity likely accelerates the development and progression of AD [29,40]. On the contrary, enhancement of Aβ-degrading enzyme activity would promote Aβ degradation [13,22]. In the present study, Xan and NAC prevented the inactivation of NEP by HNE or oligomeric Aβ_42_ treatment. *V*_max_ and *K*_m_ analyses revealed that NEP activity followed Michaelis–Menten kinetics, with a hyperbolic dependence of *v* (velocity) on substrate concentration. HNE or oligomeric Aβ_42_ decreased *V*_max_ and *K*_m_ of NEP activity, whereas Xan or NAC restored *V*_max_ and *K*_m_ of NEP activity in cells with HNE or oligomeric Aβ_42_ treatments. These results suggest that a reduction in NEP activity by HNE could be reversed by antioxidants. Furthermore, the present experiment demonstrated that Xan or NAC treatments degraded both monomeric and toxic oligomeric Aβ_42_ and protect neuronal cells against oligomeric Aβ_42_-induced toxicity via enhancing NEP activity.

Numerous studies have demonstrated that direct antioxidants, such as flavonoids, indirect antioxidants, such as NOS inhibitors, and metabolic antioxidants, such as NAC, can prevent neurodegeneration in AD [41,42]. The present results indicate that these actions are related to the protection of the Aβ-degrading enzyme NEP from oxidative modification and inactivation. Though NEP is a major physiological Aβ-degrading enzyme, several other enzymes such as angiotensin-converting enzyme also degrade Aβ peptides [9,14,15,20]. Future studies will examine roles of these Aβ-degrading enzymes in oxidative modification and Aβ-degrading activity, and effects of antioxidants on these enzymes.

However, amyloid plaque composed of Aβ is one of two major pathological features in AD. Even though if it is limited to the amyloid cascade hypothesis, aggregation of Aβ evokes oxidative damage, inflammation, and neurotoxicity. Oxidative damage contributes to inflammation in AD and Aβ-induced neurotoxicity is exacerbated under inflammation dysregulation [7, 23]. The present study proved only the possibility of Xan as an antioxidant treatment of AD. Because, as already reported, Xan has a variety of biological activities such as anti-inflammatory properties [31]. These properties and efficacies of Xan need to be studied using primary cultured neurones *in vitro* as well as *in vivo* in the brains of AD animal models with several pathological features of AD, with research to reveal its molecular and cellular mechanism.

## Abbreviations

AD: Alzheimer’s disease
Aβ: amyloid-β peptide
CCK-8: Cell Counting Kit-8
HFP: 1,1,1,3,3,3-Hexafluoro-2-propanol
HNE: 4-hydroxynonenal
HRP: horseradish peroxidase
NAC: N-acetyl-l-cysteine
NEP: neprilysin
ROS: reactive oxygen species
TP: thiorphan
Xan: xanthorrhizol
